# Reconstruction versus conservative treatment after rupture of the anterior cruciate ligament: cost effectiveness analysis

**DOI:** 10.1186/1472-6963-11-317

**Published:** 2011-11-19

**Authors:** Mazda Farshad, Christian Gerber, Dominik C Meyer, Alexander Schwab, Patricia R Blank, Thomas Szucs

**Affiliations:** 1 Department of Orthopedic Surgery, University of Zürich, Balgrist University Hospital, Zürich, Switzerland; 2 Balgrist University Hospital, Financial Management and Controlling, University of Zürich, Balgrist University Hospital, Zürich, Switzerland; 3 Institute of Pharmaceutical Medicine, University of Basel, Basel, Switzerland

## Abstract

**Background:**

The decision whether to treat conservatively or reconstruct surgically a torn anterior cruciate ligament (ACL) is an ongoing subject of debate. The high prevalence and associated public health burden of torn ACL has led to continuous efforts to determine the best therapeutic approach. A critical evaluation of benefits and expenditures of both treatment options as in a cost effectiveness analysis seems well-suited to provide valuable information for treating physicians and healthcare policymakers.

**Methods:**

A literature review identified four of 7410 searched articles providing sufficient outcome probabilities for the two treatment options for modeling. A transformation key based on the expert opinions of 25 orthopedic surgeons was used to derive utilities from available evidence. The cost data for both treatment strategies were based on average figures compiled by Orthopaedic University Hospital Balgrist and reinforced by Swiss national statistics. A decision tree was constructed to derive the cost-effectiveness of each strategy, which was then tested for robustness using Monte Carlo simulation.

**Results:**

Decision tree analysis revealed a cost effectiveness of 16,038 USD/0.78 QALY for ACL reconstruction and 15,466 USD/0.66 QALY for conservative treatment, implying an incremental cost effectiveness of 4,890 USD/QALY for ACL reconstruction. Sensitivity analysis of utilities did not change the trend.

**Conclusion:**

ACL reconstruction for reestablishment of knee stability seems cost effective in the Swiss setting based on currently available evidence. This, however, should be reinforced with randomized controlled trials comparing the two treatment strategies.

## Background

Rupture of the anterior cruciate ligament (ACL) changes the kinematics of the knee [1] and often results in instability with accompanying functional disability and pain [2-22]. Although there are more than 2000 scientific articles in the literature [23] illuminating several aspects of ACL rupture, there is no consensus on the optimal treatment. Whereas some authors reported adequate outcomes after operative treatment using various techniques [8,13,24-44], others documented sufficient clinical results after conservative treatment with various protocols of immobilization and physiotherapy [4,45-64]. Although several instruments and scoring systems [65-69] have been developed to facilitate standardized reporting and comparison of differently treated patients, decision towards one or the other, namely, conservative or surgical treatment seems currently challenging [14] due to lack of randomized controlled trials with information on long-term results [70].

Most surgeons advocate ACL reconstruction for patients with ACL rupture associated with subjective instability whereas some orthopedic surgeons routinely favor conservative treatment of ACL ruptures. Thus there is still controversy on this common injury with an estimated incidence of approximately 1500/100,000 person-years in Switzerland, 1200/100,000 person-years in New Zealand [71], and 3000/100,000 person-years in the United States [23]. The occurrence of ACL ruptures depends on sex, age, and sports activities of those affected [72]. The Swiss National Insurance System for Injuries (UVG), which covers half the Swiss population, provides around 200-250 million US dollars equivalent yearly for patients with ACL injuries, including 40% of direct treatment costs.

A critical evaluation of benefits and expenditures of the two treatment options so as to provide valuable information for treating physicians and healthcare policymakers is in progress. Technical arguments appear unable to determine superiority of one or the other strategy and complementary research using economic and public health approaches including assessment of quality of life, direct cost, and cost effectiveness is necessary. Although cost effectiveness would significantly affect the decision toward one or other strategy, such studies for this common injury are rare [73,74]. A cost effectiveness analysis would allow rational allocation of limited resources and resolve an uncertainty that might potentially have been created by setting the focus on purely medical factors rather than economics aspects.

Gottlob et al [75] reported that in young adults in the United States, surgical treatment of ACL ruptures was more cost-effective than conservative treatment. However, due to lack of studies comparing the two treatment options in the same study groups at that time (1999) as well as more recent advances particularly in the surgical treatment of ACL ruptures, the results must be interpreted with caution and might not represent the current status. Although several authors aimed to compare surgical with conservative treatment [2-22], these reports are difficult to use for cost effectiveness analysis due to lack of necessary information and use of outdated surgical techniques. The purpose of the present cost-utility analysis was to identify the more cost effective treatment option for ACL ruptures in patients at an average age 30-35 years from the viewpoint of third party payers in the Swiss setting by the use of evidence created by studies comparing directly both treatment options in the same study population.

## Methods

### Literature review and extraction of effectiveness data

**Figure 1:**
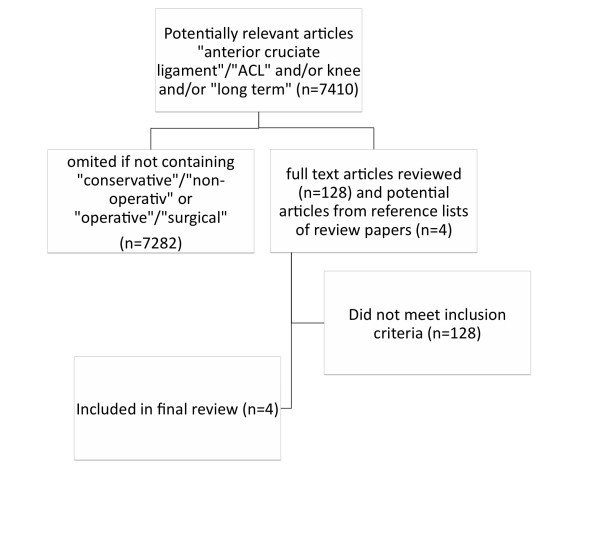
**Reviewing process of currently available literature to filter studies with direct comparison of conservative and surgical treatment and sufficient information on activity levels**.

We retrieved 7076 articles in Medline by searching with the keywords "anterior cruciate ligament" and "knee" screening in "any field" and/or "long term" in "any field" without limitation of the study type. From those articles further search by the keywords "conservative" or "non operative" and "surgical" or "operative" identified 128 articles. Each article was screened and excluded if conservative treatment and ACL reconstruction were not directly compared, no data could be abstracted for conversion of reported outcomes to the activity score, the population of investigation comprised children or adolescents, and if the technique for surgical repair was neither semitendinous tendon grafting nor bone patellar tendon bone autograft. Additionally, reviews [10,14,50,74] were screened; four additional articles [4,11,17,21] were identified as articles comparing conservative versus operative treatment. After application of the exclusion criteria, four articles [5,7,13,19] were used for further analysis (Figure 1).

### Compilation of available evidence and transformation to utility

To assess the utilities based on activity levels as suggested and validated by Gottlob et al clinical vignettes have been designed to match Gottlob's levels of activity as I-IV, where I is a patient with symptomatic activities of daily living (ADL), II someone who can perform ADLs without symptoms but no sports, III a patient with knee symptoms when performing mildly stressful sports such as jogging, swimming, and cycling, and IV someone with knee symptoms when doing moderately stressful sports activities such as baseball, alpine skiing, and dance. Activity levels 0 and V were assumed to score lowest and highest, respectively, on all scores. Four hypothetical patients were constructed based on different levels of activities after rupture of the ACL to simulate typical situations seen in daily practice. A questionnaire including the Health Utility Index (HUI)-III for utility values, IKDC subjective score, and Lysholm and Tegner score was created and 25 orthopedic surgeons were asked to fill the questionnaire for each hypothetical patient as proxies for all patients they had treated and who would fit in that hypothetical scenario. The opinions of the surgeons were weighted on their experience with ACL patients for analysis. A transformation key was developed to transform the reported outcomes from the available studies to utility values and utility values were assigned for corresponding activity levels 0 to V as described in detail elsewhere [76]. The mean age and follow-up length of the abstracted confined population of the four retracted articles were calculated weighted according to the sample size (Table 1).

**Table 1:** Distribution of level of activities of a constructed population based on available studies after either operative or conservative treatment of torn ACL.

				Activity level (Gottlob et al)				
	Patients with activity data (n)	Age (years)	Follow Up (months)	Class I (%)	Class II (%)	Class III (%)	Class IV (%)	Class V (%)
***operative***								
Finke et al (2001)	46	34	132	2	12	12	20	0
Diekstall et al (1999)	60	27.9	51	5	6	6	11	32
Kessler et al (2008)	60	30.7	140	5	6	6	11	32
Seitz et al (1994)	63	25	102	0	0	7	56	0
mean/Sum	229	28	89	12	24	31	98	64
**%**				**5.2**	**10.5**	**13.5**	**42.8**	**27.9**
***conservative***								
Finke et al (2001)	25	32	140	11	6.5	6.5	1	0
Diekstall et al (1999)	49	23.8	53	7	7.5	7.5	20	7
Kessler et al (2008)	60	30.7	140	5	6	6	11	32
Seitz et al (1994)	21	28	102	0	3	12	6	0
mean/Sum	155	27	90	23	23	32	38	39
**%**				**14.8**	**14.8**	**20.6**	**24.5**	**25.2**

### Cost data

Cost data were based on average cost of treating patients with ACL ruptures at the Department of Orthopedic Surgery, Orthopaedic University Hospital Balgrist (University of Zürich, Switzerland). Hospitalization data were analyzed for 254 consecutive patients who underwent ACL reconstruction between 2005 and 2009. From those, the last 31 consecutive cases representing the cohort of 2009 were analyzed in detail. The data of the remaining 223 patients (2005-08) were used to assure that the cohort of 2009 was representative. For the outpatient portion of the treatment before and after ACL reconstruction and for conservative treatment the experts were asked to assess what kind of resources the average patient with torn ACL experiencing joint instability would use in what frequency. Both used resources and prices per unit of each resource were extracted from detailed cost statistics provided by Orthopaedic University Hospital Balgrist. Furthermore, experts were asked to provide names of typical patients for each of the surgical and conservative arm to validate the calculations that were derived from statistical analysis. In addition, cost data by the Swiss National Insurance for Accidents (UVG) were used to confirm the ability of our cost data to represent the average Swiss patient undergoing either surgical or conservative treatment for ACL rupture.

Direct costs of potential long-term complications after ACL rupture, namely, meniscal lesions and osteoarthritis were calculated based on the analysis of 73 and 406 consecutive patients who underwent treatment for meniscus lesions (3847 USD) or osteoarthritis (total knee prosthesis, 14,826 USD) during 2006-09, which were however not necessarily identified as direct complications of ACL rupture and inpatient costs only. The perioperative costs were assumed in the same range as for ACL surgery (2535 USD) (Table 2) and were added to the in-hospital costs for each complication.

**Table 2:** Total direct costs of operative and conservative treatment of a torn ACL.

	Resource	amount	costs (USD)	costs per unit (USD)
**Surgical treatment**	Outpatient visit (15 min)	5(2-6)	718	144
	Xray (Knee, 3 views)	1(2-3)	128	128
	MRI	1	419	419
	In-hospital stay and OR	4.8 days	7391	7391
	*Medication ambulant*			
	low molecular heparin	16 days 1/day	150	9
	analgesic agents	16 days, 3/day	157	3
	Physiotherapy units	14 (9-27)	672	42
	Orthesis	1	291	291
	**Total**		**9926**	
**Conservative treatment**	Outpatient visit (15 min)	3(2-10)	431	144
	X-ray (Knee, 3 views)	1(1-2)	128	128
	MRI	1	419	419
	*Medication*			
	low molecular heparin	21 days, 1/day	197	9
	analgesic agents	21 days, 3/day	205	3
	Physiotherapy units	18 (9-27)	864	42
	Orthesis	1	291	291
	**total**		**2535**	

Prices per unit are documented in USD calculated by conversion of Swiss Francs (CHF) by a factor of 1.15 based on the exchange rate as of May 3, 2010.

### Modeling

A decision tree was constructed using the software Treeage Pro 2009 over a time horizon of 90 months based on the constructed study population (Table 1). In the surgical arm, patients undergoing ACL reconstruction without complications (osteoarthritis or meniscal lesions) were distributed to activity levels 0-V on the basis of currently available evidence (Table 1). The corresponding utility values[76] were used for each class of activity. In 3.5% of patients, ACL-reconstruction failed and re-reconstruction was needed. For those patients the activity level was assumed one class lower than before failure, except patients in class 1 remained in the same class. The same approach was used for construction of the conservative arm with the according probabilities and utility value for each activity class. In the model 16% of conservatively treated patients required surgical ACL reconstruction. Other studies have shown even higher (up to 39%) need for ACL reconstruction in conservatively treated patients [70]. The average costs of the surgical arm without complication were used for patients added to the costs of initial conservative therapy.

The probability of sequelae associated with ACL rupture for patients after ACL reconstruction was set at 34% on the basis of the retracted articles that could provide sufficient long-term information [8,19]. From those 34%, the major fraction was osteoarthritis (86%) followed by meniscal lesions (14%). For patients who decided to undergo conservative treatment, the probability of developing complications was higher (77%). Their ratio of osteoarthritis to meniscal lesions, however, was not the same (74% and 26%). It was assumed that all complications needed to undergo surgical therapy and the costs of surgical therapy for meniscal lesions and osteoarthritis were added to those of ACL repair or conservative treatment in such cases. Patients with sequelae of each treatment method were assumed in activity class II.

Sensitivity analysis was performed to test the robustness of the model. The uncertainty for the assigned utility values to the activity classes was tested for robustness by Monte Carlo probabilistic sensitivity analysis by using 10,000 sets of parameter values randomly sampled from a normal distribution (normal distribution and standard deviations (SD) as gained from the literature[76]). Parameters covered included all utility values. Furthermore, the incremental cost effectiveness was calculated for the worst-case assumption where no attention would be given to complications such as meniscal lesions and osteoarthritis.

## Results

### Extraction of effectiveness data and compilation to utility

The available literature was sufficient to allow construction of a population of 384 patients (229 treated surgically and 155 conservatively) with a mean follow-up after surgical and conservative treatment of 89 months and 90 months, respectively. Using the transformation key based on the experts survey[76], level of activities could be assigned to patient groups of extracted articles (Table 1). The proportion of patients with high levels of activity (IV and V) was higher after surgical (70.7%) than conservative treatment (49.7%) (Table 1).

### Costs

Direct costs were higher in surgically (9926 USD) than in conservatively treated patients (2535 USD). The main contributor to the cost of ACL reconstruction was in-hospital stay with a mean of 4.8 days (7391 USD) (Table 2). The costs of ACL reconstruction extracted and analyzed on the basis of data compiled by Orthopaedic University Hospital Balgrist overestimated as expected the values provided by the Swiss UVG, with 8673 CHF (7536 USD) for both surgically and conservatively-treated patients.

### Cost effectiveness

Decision tree analysis revealed a rate of 16,038 USD/0.78 QALY for ACL reconstruction and 15,466 USD/0.66 QALY for conservative treatment, implying a cost effectiveness for the two treatments of 20,612 USD/QALY (SD: 1941 USD/QALY) and 23,391 USD/QALY (SD: 5603 USD/QALY), respectively, and an incremental cost effectiveness (the incremental cost divided by the incremental effectiveness, not the difference of cost effectiveness of one minus the other strategy) of 4890 USD/QALY for ACL reconstruction (Table 3).

**Table 3:** Incremental cost effectiveness analysis for reconstructive therapy of torn ACL.

Strategy	Cost	Incremental Cost	Effect	Incremental Effect	Cost Effectiveness	Incremental Cost Effectiveness
Conservative	USD 15466		QALY 0,66		USD/QALY 23391	
Reconstruction	USD 16038	USD 572	QALY 0,78	QALY 0,12	USD/QALY 20612	**USD/QALY 4890**

**Figures 2:**
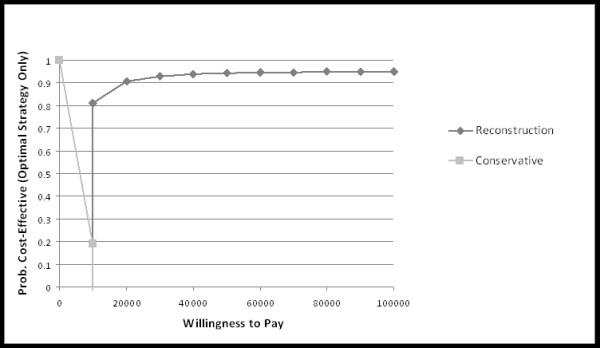
**The cost-effectiveness acceptability frontier shows the probabilistic sensitivity analysis-based on the probability of surgically and conservatively treated ACL patients of being cost-effective**. For different willingness to pay thresholds, a different strategy is preferred. For each threshold, only the probability for the optimal strategy is shown.

**Figure 3:**
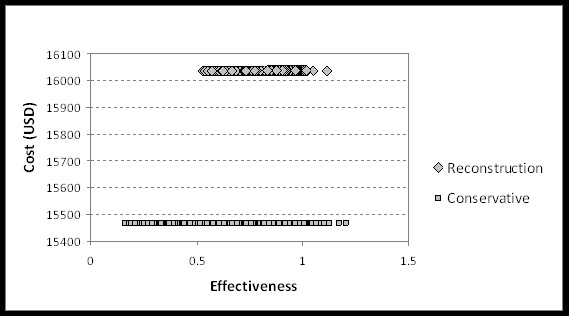
**The cost-effectiveness scatter plot uses the cost-effectiveness plane to plot the cost and effectiveness pair for each recalculation of the model with 10,000 runs for each strategy**.

Sensitivity analysis by Monte Carlo probabilistic simulation for simultaneously varying utility values documented 16,038 USD with a utility value of 0.78 ± 0.07 for surgical therapy and 15,466 USD with 0.66 ± 0.13 for conservative therapy according to a cost effectiveness of 20,687 ± 1959 USD/QALY and 24,467 ± 5656 USD/QALY, respectively. At a willingness to pay/QALY of 10000 USD the reconstruction became the preferred strategy (Figure 2). Additional probabilistic sensitivity analyses are shown in Figure 3.

In the worst-case scenario, not accounting for sequelae such as late meniscal lesions or development of osteoarthritis, the incremental cost effectiveness would be 68,715 USD/QALY for surgical treatment.

## Discussion

The decision whether to treat conservatively or reconstruct surgically a torn ACL has been debated throughout the history of knee surgery. The high prevalence and associated public health burden of torn ACL has led to continuous arguments in favor of one or the other strategy, which produced, however, no clear solution. Although thousands of studies have been published in regard to ACL [23], a critical evaluation of benefits and expenditures of both treatment options to provide valuable information for treating physicians and healthcare policymakers has not been performed [74]. Here, we analyzed the cost effectiveness of the two procedures in the Swiss setting and found surgical reconstruction to be cost effective assuming the patient has from symptoms such as the knee giving way, pain, or instability.

The results of our analysis must, however, be interpreted with caution. First, the information of clinical outcome or effectiveness for each treatment approach was based on compiled data from reported studies. Efforts were made to review systematically the currently available literature (Figure 1) so as to find the most suitable sources of information. Although the retracted studies [5,7,13,19] were potentially heterogeneous and were not randomized controlled trials, they did compare the two treatment strategies in the same experimental setting and provide sufficient outcome data for abstraction to utility values.

Second, the decision tree is a model only. On the other hand, sensitivity analysis showed a very robust model. The most sensitive determinant changing the incremental cost effectiveness for surgical therapy > 10 fold to 68,715 USD/QALY was removal of sequelae of torn ACL. This however is an unrealistic scenario [7,19]. Changes in the kinematics of gait produced by a deficient ACL have been described to result in subsequent osteoarthritis relatively unrelated to whether a reconstruction has been performed [77,78]. Meniscal lesions are commonly concomitant to ACL ruptures and also play a contributive role in development of osteoarthritis [79,80]. ACL-deficient, conservatively treated patients do need more often surgical treatment for meniscal lesions [5,7,13,19]. The results of studies that might describe no difference in sequelae for either treatment strategy should be interpreted with caution to a common limitation being a selection bias of patients with less severe injuries to the conservative arm of the study. Further, the severity of osteoarthritis should be considered in studying long-term results of both treatment options; while the overall rate of osteoarthritis might not significantly be related to the treatment procedure, more severe degeneration has been reported in patients undergoing conservative treatment [81]. It is however unquestionable that some patients will benefit more from ACL reconstruction than others. How and when to select patients for surgery remain strongly disputed issues. Stratification regarding the need for surgery has not been possible in the current analysis because there are no uniform guidelines or consensus.

Third, the cost of the conservative arm seems underestimated. Although hospital infrastructure, administration, and organization costs were mainly covered by surgically treated patients with in-hospital stays, these are also significantly used by ambulatory patients such as those whose ACL is conservatively treated.

## Conclusion

ACL reconstruction is cost effective. Our calculated incremental cost effectiveness of 4890 USD/QALY is in good agreement with the hitherto only available analysis performed by Gottlob et al (5857 USD/QALY) [74]. However, although the results of this study might contribute to informed decision making for health policymakers, the individual situation of the patient must be respected when suggesting one or the other strategy.

## Competing interests

The authors declare that they have no competing interests.

## Authors' contributions

All authors have made substantial contributions to this study; MF was involved in conception and design and the acquisition of data, analysis and interpretation of data and in drafting the manuscript. GC was involved in conception and design and the interpretation of data, revising the manuscript critically for important intellectual content and supervision. DCM was involved in conception and design and the acquisition of data, analysis and interpretation of data and in drafting the manuscript. AS was involved in the acquisition of data, interpretation of data and revising the manuscript critically for important intellectual content. PB was involved in analysis and interpretation of data and revising the manuscript critically for important intellectual content. TS was involved in conception and design and interpretation of data and revising the manuscript critically for important intellectual content and supervision. All authors read and approved the final manuscript.

## Pre-publication history

The pre-publication history for this paper can be accessed here:

http://www.biomedcentral.com/1472-6963/11/317/prepub
